# Managing diabetes and its comorbidity: a challenge for primary care settings

**DOI:** 10.1186/1753-6561-9-S1-A63

**Published:** 2015-01-14

**Authors:** S Pati, GS Francois

**Affiliations:** 1 VU University Amsterdam, Amsterdam, Netherlands; 2 Indian Institute of Public Health Bhubaneswar, Bhubaneshwar, OD, India; 3 NIVEL, Amsterdam, Netherlands

## Background

Diabetes is a major health care challenge in India [1]. Majority of diabetics depend on primary care settings for the management of their condition. Management of diabetes could be challenging for primary care provider owing to its co morbidities. Present exploratory study assessed the availability of resources at the primary health care facilities in Odisha, for managing diabetes as well as explored primary care physicians challenges and constraints in managing this condition.

## Methods

Thirty primary care centres in Odisha (ten from urban, semi urban and rural each) were randomly selected. Evaluation of facilities provided at their level was assessed by a modified version of PCET(Primary Care Evaluation Tool) [2] and descriptive statistics was computed. Additionally two Focus Group Discussion with 12 physicians of the study group was done using Thematic framework approach.

## Results

It was found that majority of centres attend to more than 5000 patient population (28 out of 30). Though all of them attended to diabetic patients in their practice area, none had special diabetics clinics. Majority (28 out of 30) made use of clinical guidelines in their practice. However with their record keeping system most of them were unable to generate a list of diabetics in their practice area (21 out of 30).Availability of IEC material was quite low (3 out of 30).None of the centres had physiotherapist or nutritionist but majority (28 out of 30) had a pharmacist who only dispensed medicines. Equipments for basic tests like blood sugar estimation were available in 5 centres. None had opthalmoscope, X-Ray facilities or USG facilities. Only two had provision of oral hypoglycaemics. None had the system of immunization for diabetics. The FCD highlighted the constraints of physicians in diabetes management. Most diabetics were referred to higher centre due to inadequate laboratory services. Huge burden of consultations per day, led to shorter consultation time for evaluation and co morbidity management. Emphasis was laid on the need for CME and lucid clinical guidelines. It was also felt that low patient awareness and loss to follow up were a hindrance.

## Conclusions

The primary care facilities need better resources and logistic support for management of diabetes and its complications. There is also a need for the capacity building of doctors through CME and IEC material which could be made available in these centres. Lack of human resources, laboratory facilities was major constraints. There is need for STP for managing diabetes and co morbidities specially conceptualised for primary care settings.
